# Microstructural and Mechanical Properties of Binary Ti-Rich Fe–Ti, Al-Rich Fe–Al, and Ti–Al Alloys

**DOI:** 10.3390/ma12030433

**Published:** 2019-01-31

**Authors:** Daoud Chanbi, Leïla Adnane Amara, Erick Ogam, Sif Eddine Amara, Zine El Abiddine Fellah

**Affiliations:** 1 Laboratoire d’Electrochimie, Corrosion, Métallurgie et Chimie Minérale, Université des Sciences et de la technologie de Houari Boumediene, BP 32 El Alia Bab Ezzouar, Algiers 16111, Algeria; daoudchanbi@gmail.com (D.C.); l_adnane@yahoo.fr (L.A.A.); samara@usthb.dz (S.E.A.); 2 Laboratoire de Mécanique et d’Acoustique, LMA-UMR 7031 Aix-Marseille University-CNRS-Centrale Marseille, F-13453 Marseille CEDEX 13, France; fellah@lma.cnrs-mrs.fr

**Keywords:** Ti–Fe, Ti–Al, Fe–Al, alloys, microstructure, intermetallic, elastic constants, Resonant Ultrasonic Vibration (RUV)

## Abstract

Three series of binary, FeTi (Ti-rich), FeAl and TiAl (Al-rich) alloy samples were produced in an argon arc furnace. An annealing treatment of 72 h at 1000 °C was applied to the samples, giving rise to different equilibrium microstructures depending on chemical composition. Their mechanical properties were studied through the determination of elastic constants that measure the stiffness of the elaborated materials. Young’s modulus of the binary alloys was determined using Resonance Ultrasonic Vibration (RUV). The accuracy of this technique was demonstrated. A scanning electron microscope (SEM) with an energy dispersive spectrometer (EDS) and X-ray diffraction (XRD) made it possible to identify intermetallic compounds FeTi and ${\mathrm{Fe}}_{2}$Ti, FeAl and Fe${\mathrm{Al}}_{2}$, and TiAl and Ti${\mathrm{Al}}_{2}$ in respective systems Fe–Ti, Fe–Al, and Ti–Al. The link between their composition, microstructure, and elastic properties was established.

## 1. Introduction

The development of the intermetallic alloys Ti–Al, Fe–Al and Fe–Ti is essentially related to the properties and performance of these materials compared to steel’s constituent pure elements, namely, iron, titanium, aluminum, and for some uses, nickel-based superalloys. Indeed, economic reasons (reduction of costs) and industrial reasons (reduction of material density or environmental nuisances) explain why they are highly sought after by certain industries, in particular, aeronautics industries. Today, they are used for properties such as friction resistance, hardness, and high-temperature and corrosion resistance [1,2,3]. As a result, Ti–Al alloys are potential materials for the aerospace, chemical, and energy industries [4,5]. Fe–Ti alloys have excellent resistance to friction, and are widely used in the automotive industry [6]. Fe–Ti and Fe–Al alloys have particular advantages: they are very affordable raw materials, and they have low density, excellent corrosion resistance, even in aggressive environments, and good mechanical properties [7,8].

The influence of different microstructures on macroscopic mechanical properties has been the subject of numerous studies [9,10,11]. Ti–Al alloys have a fully lamellar microstructure and offer a good balance between mechanical properties such as elasticity at room temperature [4,12]. The mechanical properties of Fe–Al and Fe–Ti alloys are not only influenced by the chemical composition contained in the alloy, but also by the dendritic microstructure formed in intermetallic alloys [13]. The main microstructural constituent formed during the solidification process of single- or dual-phase Fe-based intermetallic alloys is the dendritic microstructure [14]. The dendritic microstructure plays an important role in giving better mechanical properties, such as elasticity and hardness [6]. This study focuses on the effect of alloy microstructures on the mechanical properties. The microstructure is strongly linked to the mechanical properties; therefore, its identification and the determination of its influence on the different phases and on the phase diagrams are important [15]. The literature reports a number of studies where the Young’s modulus of the studied alloys in the present work was experimentally determined using nanoindentation, and very few using ultrasonic vibration (see Table 1).

Studies linking the microstructure and Young’s modulus obtained using acoustic methods are scarce.

Many theoretical and experimental methods are available to evaluate elastic constants, including the ab initio method used to theoretically predict mechanical properties. The ab initio method is based on density functional theory (DFT), which is used to perform quantum calculations of the solid’s electronic structure. This is the approach that was employed to explore the relationship between chemical composition and elastic properties. Experimentally, the Resonant Ultrasonic Vibration (RUV) method was developed in this study and used to recover Young’s modulus of the alloys. These constants can be easily calculated knowing Hooke’s law [22,23]. A variant of the method developed in this study is Resonant Ultrasound spectroscopy (RUS), which is an accurate and efficient method for characterizing the elasticity of isotropic or anisotropic solid materials [24]. RUS often involves the use of shear-wave type piezoelectric transducers (PZT) making dry-point contact with the sample’s corners (corners are used because they provide elastically weak coupling, reducing loading). However, sample sizes in RUS are often very small (typically between 1.0 mm and 1.0 cm in size). The Lagrangian minimization (the Rayleigh–Ritz variational method) is the most used in RUS analyses for forward problems [22,23].

In this part of our work, the objective was to identify the formed phases in the series of binary alloys being investigated and understand their solidification microstructures (their phase distribution, compositions, and morphologies). This allows control of solidification and the industrial development of viable alloys, together with the determination of their mechanical properties and of the effect of the microstructure on their elasticity, the elastic properties, that is, Young’s modulus here. These binary alloys guide the choice of ternary grades that might be promising solutions to overcoming problems arising during the development or use of industrial alloy Fe–Al or intermetallic base Ti–Al. The effect of the chemical composition on Young’s modulus was also highlighted.

## 2. Materials and Methods

The set of binary alloys, Ti–Fe, Ti–Al and Fe–Al, were synthesized using pure metals (Fe → 99.98%, Ti → 99.99%, Al → 99.98%), in an arc furnace under argon flow. The purity of the argon (Ar) gas used to prepare the alloys was 99.9999%. In order to ensure good homogeneity of the samples, the fusion process was repeated four times for each. Verification of the nominal composition after elaboration was performed using X-ray fluorescence spectrometry (XRF) (Horiba XGT-5000, Kyoto, Japan). This XRF apparatus typically covers all elements, from Sodium (Na) to Uranium (U), (Z Na = 11 to Z U = 92). Each chemical element present in the alloy, ranging from Na to U is characterized by two essential peaks, Kα and Kβ. The Kα peak is more intense than the Kβ peak. The intensity of each characteristic radiation is directly related to the amount of each element in the material. Once composition was verified, our alloys were annealed for 72 h at 1000 °C (heating rate 20 °C/min) and then cooled (slow cooling rate of 5 °C/min), which gave them their physicochemical, mechanical, and structural balance. Thus, the obtained structure is that reported on the equilibrium diagrams at room temperature (part of another study). They were then prepared and polished for observation. This was achieved using a scanning electron microscope coupled with a dispersive energy spectrometer (SEM-EDS) (Fei QUANTA 600-EDAX, Hillsboro, Oregon, USA). An X-ray diffractometer (Bruker D8 Advance, Billerica, MA, USA) was used to identify the formed phases using the following parameters: voltage 40 keV, intensity 30 mA, Ω=2θ, and λCuKα = 1.54056 Å.

## 3. Preparation of Disc Surfaces for Mechanical Characterization

Prior to experimental analysis, the disc samples were first shaped so that the diameter-to-thickness ratio was approximately 4 (for simplicity of analysis of the experimental data). They were then polished using sheets of sandpaper with different grit sizes (240, 400, 600, 800, and 1200), resulting in well-polished surfaces and well-adjusted sample sizes, with a diameter of 14 ± 0.5 mm and thickness of 3 ± 0.5 mm.

### Configuration with Piezoelectric Discs

**Figure 1:**
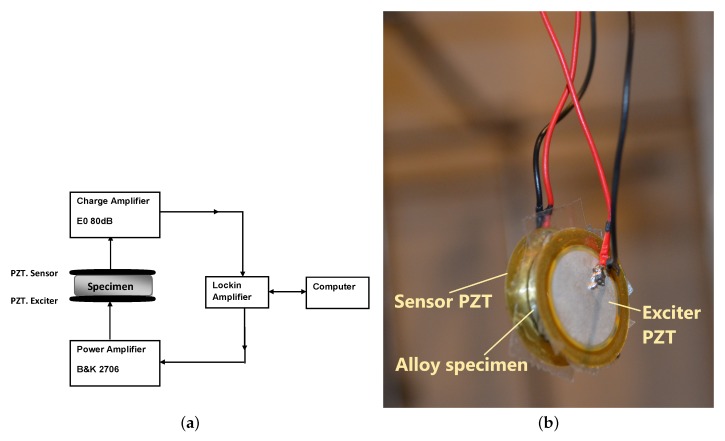
(**a**) Diagram and (**b**) photography of the setup employed for the vibration spectroscopy experimental setup.

The configuration employed two thin piezoelectric transducer (PZT) discs. The PZT discs were retrieved from the resonators of the piezoelectric audio transducers used as sounders. Since their response characteristics were not known beforehand, three different discs were tested on the specimens and their responses evaluated to obtain the best choice for the experiment. They were referenced as KPEG827, KPEG110, KPEG165 (Kingstate, Taipei, Taiwan), with diameters of 25, 20, and 10 mm, respectively. Two thin PZT discs were necessary and had the same diameter. The first PZT disc was used as the exciter and the second as sensor to capture the spectral vibration response of the metal sample. The carefully polished metal-disc alloy sample was placed between the pair of PZT transducer discs. The PZT disc, Kingstate KPEG 110, 20 mm diameter, gave the best response for the tested samples. The experimental setup diagram and photograph are shown in Figure 1. A lock amplifier (Signal Recovery 7265 DSP, Oak Ridge, TN, USA) was employed to drive the exciter in discrete frequency steps. Thin double-sided adhesive tape was used to assure good contact between the two flat surfaces of the sample and the PZT. The PZT disc and sample taped to it were then suspended to ensure stressfree vibrations, i.e., without constraint (Figure 1). The adhesive tape was cut to exactly fit PZT-disc size in order to limit the impact of damping on the measurements that the tape may add. The sample was then excited using continuous harmonic stimulation, realizing a frequency sweep (frequency, 50–250 kHz in 200 Hz steps). The response was captured by the second piezoelectric disc connected to a low-noise amplifier (EO80dB, Ciprian, Grenoble, France). Rotation of the alloy-disc sample in several directions was performed to confirm the similarity of vibration response spectra, verify the isotropy of the samples, and confirm the repeatability and consistency of the obtained experimental data.

## 4. Ingredients for Estimating Alloy-Disc-Sample Elastic Modulus

To simplify mechanical behavior analysis, the alloy samples in this study were considered as equivalent single-crystal elastic bodies. Their mechanical behavior was accurately captured using the elastodynamic model of an anisotropic elastic solid. Two elastodynamic models of vibration were candidates for solving the direct problem; the first incorporated more physical and geometric information on the sample, but required more intensive computations. The second model was not computationally intensive and was chosen as a candidate to solve the inverse problem, which was the recovery of Young’s modulus and Poisson ratio from the resonance frequencies of the samples. The second model was validated using the first model. The first model was then used to finely adjust the two parameters that had been identified through solving the inverse problem.

### 4.1. First Model for Solving Direct Problem

The choice of the correct model for solving the direct problem is a key issue for the validation of data retrieved from experimental measurements. The inversion process can be one in which a set of computed observables, in this case, eigenfrequencies, are used to calculate a succession of trial values of the sought for parameters of the configuration from experimental data. In such a process, there is need to compute the resonance frequencies of a given structure configuration and then compare them with the measured resonance frequencies of the test sample to recover its elastic modulus and Poisson ratio. In this study, the computed eigenfrequencies were obtained using an interaction model based on the finite element method. They were then compared with the experimental resonance frequencies recovered from the measured spectral response. This permitted the retrieval of the optimal values of the elastic modulus and Poisson ratio that were recovered by the second model. Precisely, the computations were done in three dimensions (3D) using an elastodynamic finite-element method (FEM) [25,26] implemented in commercial software Abaqus (version 6.14, Dassault Systèmes Simulia Corporation, Providence, RI, USA) [27]. This constituted the first interaction model.

### 4.2. Second Model for Solving Direct Problem

The resonance frequencies resulting from harmonic excitation applied to the sample, a thick circular disc, were computed using a quasianalytic model. This model was developed by Martinceck [28] and has also been adopted in the standard testing method [29]. The model equation defines the relationship between resonance frequency, and the elastic properties of the material and dimensions of the disc-shaped sample. It is written as follows:

(1) $$f_{i}=K_{i}/\left(2\pi r^{2}\right)\sqrt{\left(A/\rho t\right)}$$

where $f_{i}$ is the resonance frequency (*i* = 1, 2), $K_{i}$ is the geometry factor for the resonance frequency, *r* is the radius of the disc, disc constant $A=Et^{3}/\left[12\left(1-\nu^{2}\right)\right]$, *E* is Young’s modulus, *t* is disc thickness, ν is the Poisson ratio, and ρ is the density. In this study, the ratio t/r was equal to 0.42.

### 4.3. Solving the Inverse Problem of Vibrational Spectroscopy

The second interaction model equation was rewritten in order to retrieve Young’s modulus as a function of resonance frequency. The expression calculated from Equation (1) is rewritten as:

(2) $$E_{i}=\left(48f_{i}^{2}\pi^{2}r^{4}\left(1-\nu^{2}\right)\rho \right)/\left(K_{i}^{2}t^{2}\right)$$

This quasianalytical equation makes the inversion process instantaneous. The geometric factors computed for the ratio t/r = 0.42 are $K_{1}$ = 4.6356 and $K_{2}$ = 7.3284. Poisson ratio read from nomograms [28] in terms of ratio t/r = 0.4 and resonance-frequency ratio $f_{2}/f_{1}=1.57$ (for most alloys in this study) was ν=0.27. The Poisson ratio was initialized using this value. Once Young’s modulus and Poisson ratio were recovered, they were injected into the sophisticated 3D FEM to calculate the resonance frequencies, which were then compared to the measured frequencies. The values of Young’s moduli and Poisson ratios were further adjusted so that the RUV experimental frequencies and those generated by the 3D FEM matched. These were then the parameter values considered as final.

### 4.4. Identification and Classification of Vibration Modes in the Response Spectrum

A simple argument was employed to resolve the issue of identifying the modes of vibration corresponding to the observed spectral vibration peaks. The elastic properties of an alloy depend on the crystallographic structure, which depends on the chemical composition of the alloy (see Table 4). The eigenvalues of one of the components of the metallic alloy of the disc sample composed of single crystals were computed. These provided orientation where the first, second, and third modes would be situated in the vibration spectrum. The elastic moduli of the single-crystal components from ab initio computation for pure metals that make up the alloy, and parameters such as density and Poisson ratio, were employed. The values for the single crystal were from the literature; therefore, computations were completed for confirmation and to evaluate the different first-principle DFT-method calculation tools. For titanium, density functional theory using the projector augmented-wave (PAW) method in Quantum Espresso associated with the Exciting [30,31] package was employed. The mechanical parameters were then injected into the 3D finite-element model to calculate the eigenfrequencies and plot mode shapes/deformation. The identification of mode types was also theoretically confirmed. A 3D elastodynamic finite-element direct-solution steady-state dynamic analysis was used to calculate the steady-state dynamic linearized response of the disc submitted to a concentrated harmonic-force excitation [32]. The concentrated force was applied at the center of the disc to only excite longitudinal modes as confirmed in the computed response [33]. In a second step, force was applied to the edge of the disc to excite all modes. This enabled the identification of mode types (whether shear or longitudinal) captured in the vibration spectrum.

## 5. Results

### 5.1. XRF Analysis

**Figure 2:**
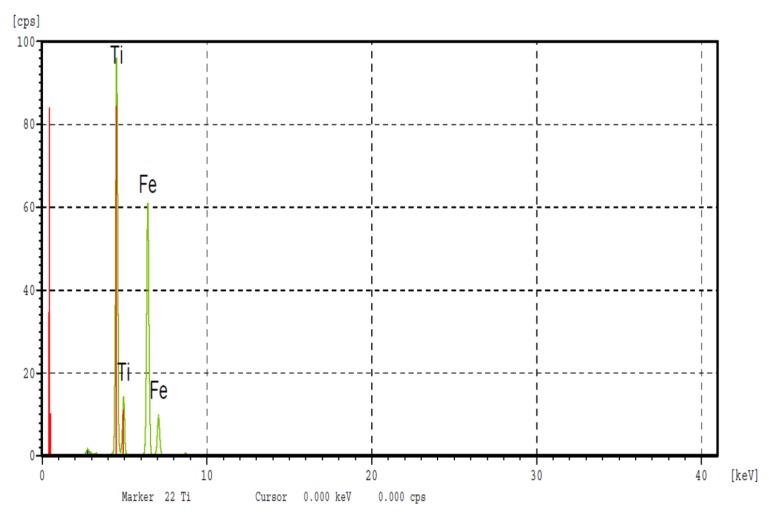
Fluorescence analysis of the XRF ${\mathrm{Fe}}_{50}{\mathrm{Ti}}_{50}$ alloy.

The XRF analysis results are listed in Table 2. The average compositions of the different binary alloys, obtained by XRF, were fairly close to the nominal compositions, as shown by the results of the analysis carried out on the ${\mathrm{Fe}}_{50}{\mathrm{Ti}}_{50}$ alloy (Figure 2).

### 5.2. Energy Dispersive Spectrometer (EDS) Analysis

The nominal chemical compositions of the various binary alloys were verified. The effective levels of Fe, Ti, and Al were provided by EDS analysis and are listed in Table 3, in which we also reported the chemical compositions of the different observed phases in each of the studied alloys here.

### 5.3. Phase Identification

**Figure 3:**
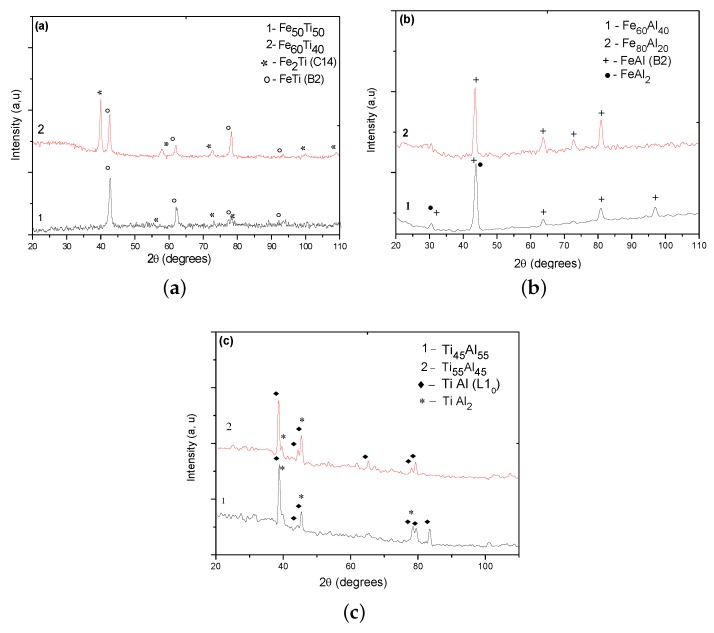
Diffractograms of the studied binary alloys: (**a**) FeTi, (**b**) FeAl, (**c**) TiAl.

The diffractograms of the various FeTi, FeAl, and TiAl binary alloys are shown in Figure 3a–c, respectively. The different identified phases, as well as the crystalline structures and their literature references for each phase, are listed in Table 4.

### 5.4. Metallography

**Figure 4:**
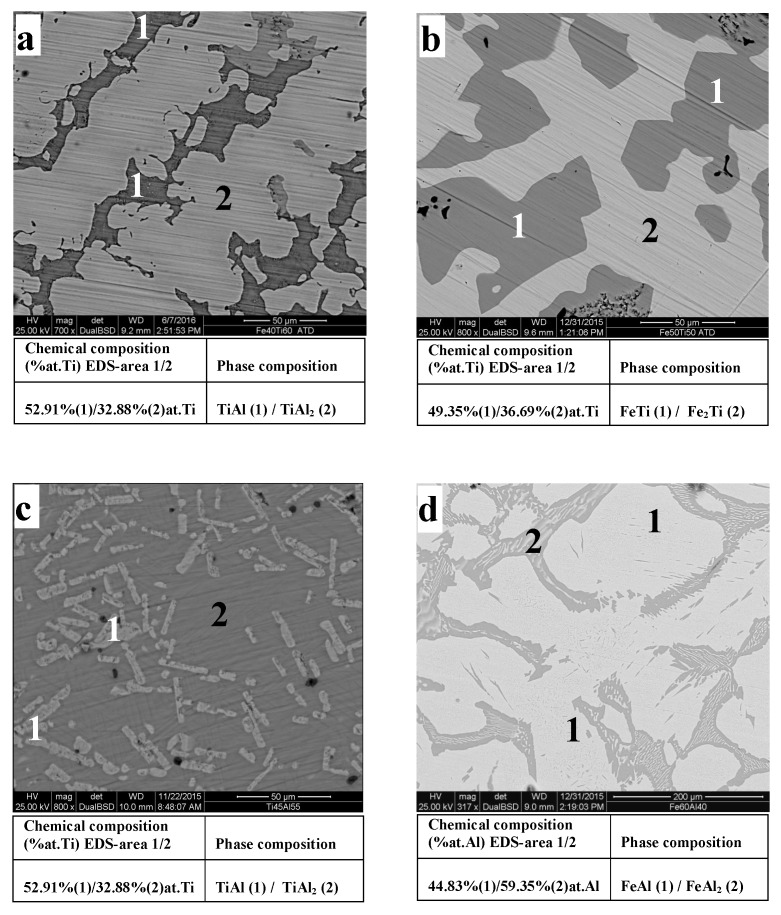
Scanning electron microscopy (SEM) micrographs of binary alloys of respective compositions (percent by weight): (**a**) ${\mathrm{Fe}}_{60}{\mathrm{Ti}}_{40}$, (**b**) ${\mathrm{Fe}}_{50}{\mathrm{Ti}}_{50}$, (**c**) ${\mathrm{Ti}}_{45}{\mathrm{Al}}_{55}$, (**d**) ${\mathrm{Fe}}_{60}{\mathrm{Al}}_{40}$.

To confirm the phases found by X-ray diffraction (XRD) analysis, we studied the morphology of the phases formed in the binary alloys (annealed at 1000 °C for 72 h at a heating rate of 20 °C/min) by scanning electron microscopy (SEM). Mcrostructures are depicted in Figure 4.

### 5.5. Vibration Modes

**Figure 5:**
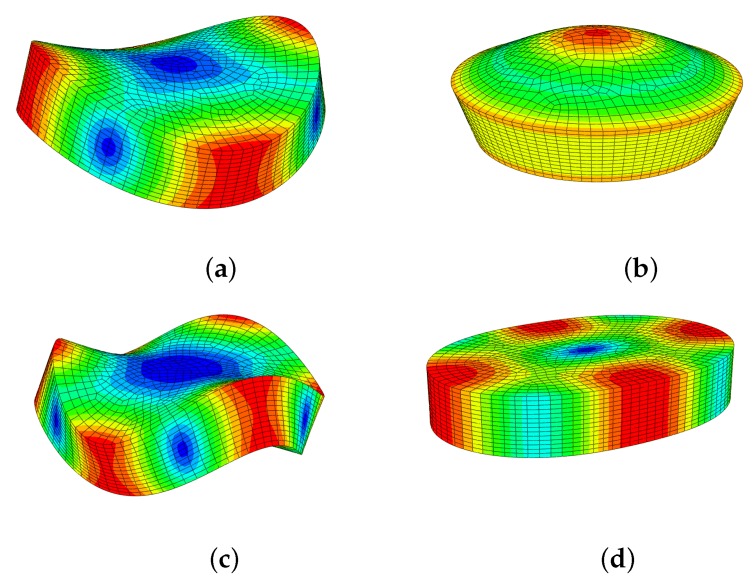
Natural vibration mode shapes of the thick discs: (**a**) first flexural mode (≈70 kHz—Table 4); (**b**) first compressional mode (≈110 kHz); (**c**) second flexural mode (≈134 kHz); (**d**) first wine-glass mode (≈165 kHz).

In order to calculate the Young’s modulus of the alloys in disc form, it was important to identify the modes of vibration to which the first and second resonance frequencies corresponded (Section 4.4), as illustrated by the mode shapes in Figure 5.

The results of the computation of the resonance frequencies of the single-crystal elements and the Young’s moduli found in the literature (from ab initio calculations) are given in Table 5. These results guided the choice of the first and second resonance family of modes from the spectral vibrational response of the alloy-disc specimens.

**Figure 6:**
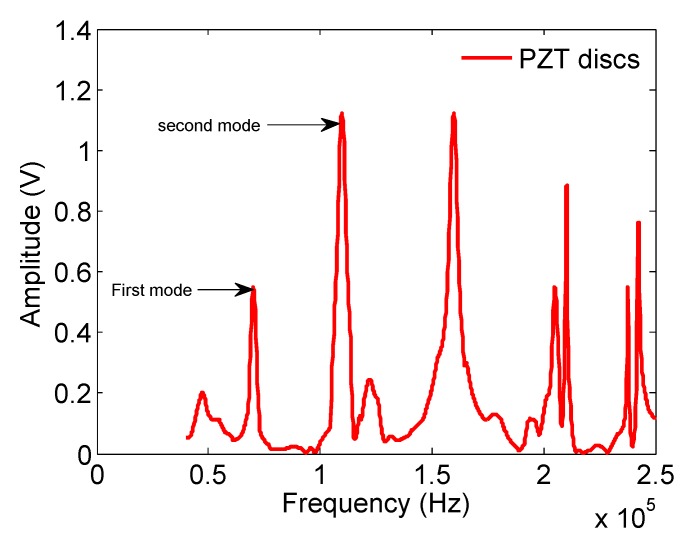
Vibration spectrum obtained using piezoelectric-disc transducers for the binary disc sample ${\mathrm{Fe}}_{50}{\mathrm{Ti}}_{50}$ composition (percentage by weight).

Figure 6 shows the positions of peaks corresponding to different modes of vibration, occurring by the excitation of piezoelectric crystals stuck onto the sample. The peaks appear sharp and well-defined with respect to the spectra obtained by the piezoelectric discs.

The final step of the inversion procedure was to obtain the elastic modulus and Poisson ratio of all alloy samples. This was achieved by minimizing the differences between the measured spectral responses and the calculated responses of the two models, which consisted of calculating the error percentages between the results of the two configuration methods as well as the experimental values obtained with the 3D finite-element method.

The recovered values of Young’s modulus and Poisson ratio obtained from calculations are shown in Table 5.

## 6. Discussion

### 6.1. Fe–Ti Binary Alloys

The diffractograms of Fe–Ti alloys (Figure 3a) show the existence of two Fe–Ti and ${\mathrm{Fe}}_{2}$Ti phases in the ${\mathrm{Fe}}_{50}{\mathrm{Ti}}_{50}$ alloy. However, only the Fe–Ti phase is revealed with certainty. Indeed, two lines of very low intensities relative to ${\mathrm{Fe}}_{2}$Ti were found in this sample, whereas, in ${\mathrm{Fe}}_{60}{\mathrm{Ti}}_{40}$, where ${\mathrm{Fe}}_{2}$Ti is unambiguously highlighted. At this stage of the investigation, only the Fe–Ti phase was clearly identified in our two samples at a time. The microstructures of the ${\mathrm{Fe}}_{60}{\mathrm{Ti}}_{40}$ and ${\mathrm{Fe}}_{50}{\mathrm{Ti}}_{50}$ binary alloys (Figure 4a,b) showed ${\mathrm{Fe}}_{2}$Ti phase dendrites surrounded by interdendritic Fe–Ti phase space. This might suggest that the Fe–Ti phase appears after the ${\mathrm{Fe}}_{2}$Ti phase. In this case, it would probably result from a peritectic-type reaction, which would then be written as L + ${\mathrm{Fe}}_{2}$Ti ↔ FeTi, as predicted by the FeTi binary diagram [43]. EDS analysis (Figure 4b) revealed the chemical compositions of each of the Fe–Ti and ${\mathrm{Fe}}_{2}$Ti phases and confirmed their formation in the two studied alloys, even if X-ray diffraction only allowed the identification of ${\mathrm{Fe}}_{2}$Ti in the ${\mathrm{Fe}}_{60}{\mathrm{Ti}}_{40}$ alloy. All of our results are in perfect agreement with what we can obtain using the Fe–Ti diagram given by Reference [44]. Moreover, our measurements of the densities of these alloys (Table 4) did not show any significant influence of the substitution of iron by titanium.

### 6.2. Fe–Al Binary Alloys

As for Fe–Al binary alloys, X-ray diffraction strongly indicated the presence of a Fe–Al phase (Figure 3b). However, some lines could be assigned to Fe–Al and Fe${\mathrm{Al}}_{2}$ at the same time, which means that we cannot ignore the presence of Fe${\mathrm{Al}}_{2}$. In Fe–Al alloys, a dendritic structure was observed thanks to EDS analysis (Figure 4d). It was identified as Fe–Al and a fine eutectic structure Fe${\mathrm{Al}}_{2}$/Fe–Al structure localised in the interdentritic space, where Fe–Al is in the form of lamellae and Fe${\mathrm{Al}}_{2}$ occupies the rest of the interdendritic space. Moreover, we could not detect the presence of the ${\mathrm{Fe}}_{3}$Al phase, widely coveted in the industry. In terms of lightening, it has been shown previously that density decreases with aluminum content [45], which was aimed at in the first place in the choice of our compositions. However, it was previously noted [46] that problems arise in the production of high-aluminum Fe–Al alloys, hence the use of vacuum or powder-metallurgy techniques. These problems include excessive metal oxidation, and the occurrence of significant internal stresses on parts, resulting in cracks and porosities. None of these problems was encountered during the development of our samples, partly because it was performed under an inert atmosphere (under argon) to prevent the evaporation of aluminum and the formation of oxides. It was also because we ensured the absence of oxide layers by stripping our metals just before shade elaboration, onto which we imposed slow cooling (rate 5 °C/min). Indeed, we, at no stage of the analysis, noted the presence of oxide in our alloys, porosity, or cracks. However, we could not rule out the possibility that a ceramic ${\mathrm{Al}}_{2}O_{3}$ film was formed on the surface of our samples, given the high aluminum content when subjected to an oxidizing medium. This layer would then have the role of improving samples’ resistance to corrosion. On the other hand, the role of iron substitution by aluminum in Fe–Al alloys is shown in Table 4, which shows that density decreased by 28% when aluminum content increased by 24%.

### 6.3. Ti–Al Binary Alloys

As for Ti–Al alloys with a high aluminum content, the formation of two Ti–Al and Ti${\mathrm{Al}}_{2}$ intermetallics in ${\mathrm{Ti}}_{55}{\mathrm{Al}}_{45}$ and ${\mathrm{Ti}}_{45}{\mathrm{Al}}_{55}$ (Figure 3b) were clearly shown. At this stage of the investigation, it seems difficult to decide on the stability of ${\mathrm{TiAl}}_{2}$ intermetallic. Indeed, this compound formed as Ti–Al in the “as-cast condition” and in those having undergone 72 h of annealing. Figure 4c is a typical microstructure of the Ti–Al alloys that we studied. It shows the existence of a two-phase structure, composed of Ti–Al lamellae in a ${\mathrm{TiAl}}_{2}$ matrix, itself lamellar, which has not been observed before. It is important to point out that a Ti–Al structure is highly dependent on the heat treatment of our alloys. In previous work [47] on aluminum-rich Ti–Al alloys, two types of duplex or lamellar Ti–Al and Ti${\mathrm{Al}}_{2}$ (stable) microstructures were obtained following the appropriate heat treatment. In crude-cast alloys, precipitates of the metastable Ti${\mathrm{Al}}_{2}$ and ${\mathrm{Ti}}_{3}{\mathrm{Al}}_{5}$ phases are often observed in the Ti–Al matrix, but are transformed into Ti–Al by heating at 900 °C. In this case, we also did not note the presence of oxides, pores, or cracks in our samples. The characteristic temperatures of predicted phase transitions only agreed well with the experimentally observed ones at high temperatures [48]. In this study, it was highlighted that the total substitution of Fe by Ti strongly affects the density of the studied alloys. They became lighter compared with the previous intermetallic base alloys (Fe–Ti and Fe–Al) (Table 4). Thus, the alloys became lighter and probably improved in corrosion resistance since Ti–Al and Ti${\mathrm{Al}}_{2}$ both have a much better resistance to oxidation than ${\mathrm{Ti}}_{3}\mathrm{Al}$ [49].

### 6.4. Retrieved Young’s Modulus Values

Alloy composition and content result in microstructural adjustment and, therefore, improvement of the mechanical properties. Yield and ultimate strengths, Young’s modulus, and creep resistance are drastically enhanced, and the high-temperature limit is also expanded [48]. In this study, we focused on the microstructure and Young’s modulus.

Before starting the discussion about a mechanical property (Young’s modulus) of aluminum-rich alloys, it should be noted that very few researchers have ventured to study this type of alloy for the problems mentioned earlier regarding their elaboration. The results reported in Table 6 show that Young’s modulus values, calculated from the spectra obtained using the piezoelectric discs, are very close to the final estimated values obtained by adjusting the parameters in the 3D finite-element model. Thus, and by way of example, the Young’s modulus of the ${\mathrm{Fe}}_{80}{\mathrm{Al}}_{20}$ and ${\mathrm{Fe}}_{60}{\mathrm{Al}}_{40}$ alloys are 201.44 and 199.56 GPa for frequencies corresponding to $F_{1}$ and $F_{2}$, respectively. The error ratio in all cases does not exceed 1% (Table 6), and the values are very close to the measured Young’s modulus of 200 GPa in Reference [2]. The similarity of $E_{1}$ and $E_{2}$ confirms that frequencies $F_{1}$ and $F_{2}$ are of the same family, and also that our alloys were homogeneous. By examining the results of Fe–Al binary alloys, it can be seen that, when Al content increases, the modulus decreases. In this case, two compounds are formed; one rich in aluminum, Fe${\mathrm{Al}}_{2}$ (small volume fraction) and the other rich in iron, Fe–Al, which could possibly explain this decrease compared with pure iron and steel, since the Young’s modulus is closely related to the bonds between atoms and the atomic stack [50]. Young’s modulus values for the ${\mathrm{Ti}}_{55}{\mathrm{Al}}_{45}$ and ${\mathrm{Ti}}_{45}{\mathrm{Al}}_{55}$ alloys are, respectively, 165.92 and 163.70 GPa, compared with those of titanium and its alloys [51] and that of Ti–Al (160 GPa). One might think that Ti${\mathrm{Al}}_{2}$ also contributes to this increase given its volume fraction. However, given the scarcity of data concerning this intermetallic alloy, it is still difficult to estimate its contribution. These results show that the addition of titanium aluminum significantly increases the elastic modulus. The respective values of the Young’s modulus for ${\mathrm{Fe}}_{60}{\mathrm{Ti}}_{40}$ and ${\mathrm{Fe}}_{50}{\mathrm{Ti}}_{50}$ alloys are 191.57 and 183.47 GPa. This shows that titanium lowers the rigidity of iron. Thus, the higher the titanium content is, the more its modulus decreases. Note that all of the values obtained with the method developed herein are close to those found in the literature [18]. As a result, alloys with a dendritic to lamellar microstructure (Figure 4) have the highest Young’s modulus, which explains why the microstructure plays an important role in the elasticity of materials.

## 7. Conclusions

In this work, a series of binary alloys, Fe–Al, Ti–Al (all rich in aluminum), and Fe–Ti (titanium-rich) were developed by fusion in an argon arc furnace using pure metals, etched just before fabrication. The important precautions taken in the solidification process revealed that, for aluminum-rich alloys, the formation of oxides, porosities, and cracks, so much feared when Al content is important, was avoided. As a result, the intermetallic Ti${\mathrm{Al}}_{2}$ in TiAl alloys, whose properties are not well-known, were formed.

Fe–Ti and ${\mathrm{Fe}}_{2}$Ti, Ti–Al and Ti${\mathrm{Al}}_{2}$, Fe–Al and Fe${\mathrm{Al}}_{2}$ phases were identified, respectively, in the Fe–Ti, Ti–Al, and Fe–Al alloys, for which the microstructures were described. The density obtained for each of the alloys did not seem to be affected by the substitution of iron with titanium, but was moderately affected when aluminum replaced titanium. However, density dropped dramatically when titanium, in the presence of aluminum, replaced iron. The experimental method of Resonance Ultrasonic Vibration, combined with theoretical approaches, were used to determine Young’s modulus of binary alloys (Fe–Al, Fe–Ti, and Ti–Al) with different chemical compositions and microstructures (dendritic, lamellar). It was found that dendritic microstructures had a higher Young’s modulus than lamellar ones. These measurements also helped reveal the isotropy of the material. This conclusion was only possible because it was found that, for all tested samples, the Young’s modulus values determined using the method agreed with the modulus retrieved using resonance frequencies and 3D finite-element formulation for the isotropic elastic model.

The accuracy of the nondestructive method developed herein using piezoelectric discs has been demonstrated by comparing the recovered Young’s modulus with those from already published results, and particularly those from first-principle calculations. The method permitted to conclude that the elaborated alloy samples were homogeneous. The mastery of the elaboration process, as well as the study of the mechanical properties and corrosion resistance of these intermetallic base alloys, appeals to the optimization of the parameters in order to find the best combination of composition and properties. This provides for the possibility of applying Al-rich Ti–Al alloys, substituted for iron and/or other elements, as alloys having high performance at high temperatures. The idea of producing a Ti–Al-like microstructure was used in the second part of our current study, which is based on the results obtained from Fe–Ti, Fe–Al, and Ti–Al binary studies.

## Figures and Tables

**Table 1:** Existing mechanical (elastic modulus) data in the literature for the studied alloys and methods employed to obtain them.

Alloy	Composition (at. %)	Young’s Modulus (GPa)	Characterization Method	Reference
TiAl		154	dynamic indentation	[16]
		160–176		[5]
γ TiAl	${\mathrm{Ti}}_{44}$ ${\mathrm{Al}}_{56}$	182	Resonant ultrasound spectroscopy Rectangular parallelepiped sample, sides (a, b, c) → (4.173, 3.993, 3.260) mm	[17]
B19 TiAl		161.99 159.24	(DFT) Strain–stress method (DFT) Energy density method	[18]
FeTi		(310, 86, 74.9)- > ($c_{11}$, $c_{12}$, $c_{44}$) Elastic constants $c_{ij}$	Ultrasonic measurement method Acoustic Resonance and velocity	[19]
FeAl	Fe${\mathrm{Al}}_{40}$	205	Tensile test	[2]
FeAl	48.71Al-50.87Fe	261	PZT ultrasonic composite oscillator-cylindrical specimens 3 mm diameter, 44 mm length	[20]
	Fe${\mathrm{Al}}_{2}$	204.5	Embedded-atom method simulation	[21]

**Table 2:** Effective compositions of the studied binary alloys obtained by X-ray fluorescence (XRF) analysis.

The Studied Alloys (w%)	${\mathrm{Fe}}_{50}{\mathrm{Ti}}_{50}$	${\mathrm{Fe}}_{60}{\mathrm{Ti}}_{40}$	${\mathrm{Ti}}_{55}{\mathrm{Al}}_{45}$	${\mathrm{Ti}}_{45}{\mathrm{Al}}_{55}$	${\mathrm{Fe}}_{60}{\mathrm{Al}}_{40}$	${\mathrm{Fe}}_{80}{\mathrm{Al}}_{20}$
Effective composition (Fe, Al, Ti)	51.12–48.88	58.71–41.29	54.67–45.33	46.25–53.75	58.64–41.36	81.71–18.29

**Table 3:** Effective compositions of the studied alloys obtained by Energy Dispersive Spectrometer (EDS) analysis.

Studied Alloys (w%)	${\mathrm{Fe}}_{50}{\mathrm{Ti}}_{50}$	${\mathrm{Fe}}_{60}{\mathrm{Ti}}_{40}$	${\mathrm{Ti}}_{55}{\mathrm{Al}}_{45}$	${\mathrm{Ti}}_{45}{\mathrm{Al}}_{55}$	${\mathrm{Fe}}_{60}{\mathrm{Al}}_{40}$	${\mathrm{Fe}}_{80}{\mathrm{Al}}_{20}$
Effective composition (Fe, Al, Ti)	50.60–49.40	61.96–38.04	55.29–44.71	45.75–54.25	58.64–41.36	79.66–20.34

**Table 4:** Identified phases and corresponding crystal structures.

Alloy Studied (w%)	Phases Formed	Crystal Structure	Prototype	Space Group	Reference
${\mathrm{Fe}}_{50}{\mathrm{Ti}}_{50}$	FeTi	B2	CsCl	*Pm-3m*	[34,35,36,37]
	${\mathrm{Fe}}_{2}$Ti	C14	Mg${\mathrm{Zn}}_{2}$	$P6_{3}6$/*mmc*	
${\mathrm{Fe}}_{60}{\mathrm{Ti}}_{40}$	FeTi	B2	CsCl	*Pm-3m*	
	${\mathrm{Fe}}_{2}$Ti	C14	Mg${\mathrm{Zn}}_{2}$	$P6_{3}6$/*mmc*	
${\mathrm{Ti}}_{55}{\mathrm{Al}}_{45}$	TiAl	$L1_{0}$	AuCu	*P4/mmm*	[34,35,36,37]
	Ti${\mathrm{Al}}_{2}$	-	Hf${\mathrm{Ga}}_{2}$	*$I4_{1}$/amd*	
${\mathrm{Ti}}_{45}{\mathrm{Al}}_{55}$	TiAl	$L1_{0}$	AuCu	*P4/mmm*	
	Ti${\mathrm{Al}}_{2}$	-	Hf${\mathrm{Ga}}_{2}$	*$I4_{1}$/amd*	
${\mathrm{Fe}}_{60}{\mathrm{Al}}_{40}$	FeAl	B2	CsCl	*Pm-3m*	[34]
	Fe${\mathrm{Al}}_{2}$	-	Fe${\mathrm{Al}}_{2}$	*P1*	
${\mathrm{Fe}}_{80}{\mathrm{Al}}_{20}$	FeAl	B2	CsCl	*Pm-3m*	

**Table 5:** Mechanical properties calculated by an ab initio method (density functional theory), and resonance frequencies of single-crystal metals computed using the 3D finite-element method ($F_{1}$, $F_{2}$). $E_{1}^{r}$ and $E_{2}^{r}$ are the recovered Young’s moduli (in GPa) using synthetic resonance frequencies ($F_{1}$, $F_{2}$) and the second interaction model (Equation (2)).

Element	Density (kg/m${}^{3}$)	Young’s Modulus (GPa)	Poisson Ratio (ν)	$F_{1}$ (Hz)	$F_{2}$ (Hz)	$E_{1}^{r}$ (GPa)	$E_{2}^{r}$ (GPa)
Fe	7874 Ref. [38]	212 Ref. [39]	0.27	70,480	110,577	213.27	210.05
Ti	4500 Ref. [40]	114.6 Ref. [38,41]	0.3	67,932	109,053	111.14	114.60
Al	2707 Ref. [38]	69.3 Ref. [38,42]	0.3	68,230	109,530	67.27	69.36

**Table 6:** Resonance frequencies ($f_{1}$, $f_{2}$) recovered from vibration spectra obtained using piezoelectric-disc transducers and the corresponding Young’s moduli ($E_{1}$, $E_{2}$), retrieved using Equation (2). Final 3D finite-element method (FEM) Young’s moduli (*E*) after adjustment to fit experimental resonance frequencies. The difference between $E_{2}$ and *E* is given as a percentage. The ${}^{†}$ indicates ab initio calculated results found in the literature. Values of $K_{1}$ = 4.6356, $K_{2}$ = 7.3284, and initial ν = 0.27.

Composition (wt.%)	Composition (at.%)	$f_{1}$ (Hz)	$f_{2}$ (Hz)	Density (kg/m${}^{3}$)	$E_{1}$ (GPa)	$E_{2}$ (Gpa)	E (Gpa) 3D FEM	$F_{1}$ (Hz) 3D FEM	$F_{2}$ (Hz) 3D FEM	Difference Percentage $E_{2}$-E (%)	Previous Studies—Ref. E (GPa), ν
${\mathrm{Fe}}_{80}{\mathrm{Al}}_{20}$	${\mathrm{Fe}}_{66}{\mathrm{Al}}_{34}$	70,400	110,200	7412	200.03	196.30	200.03	70,650	110,800	1.86	200 Ref. [2]
${\mathrm{Fe}}_{60}{\mathrm{Al}}_{40}$	${\mathrm{Fe}}_{42}{\mathrm{Al}}_{58}$	82,400	130,200	5330	197.30	197.10	197.30	82,840	130,000	0.10	
${\mathrm{Fe}}_{60}{\mathrm{Ti}}_{40}$	${\mathrm{Fe}}_{56}{\mathrm{Ti}}_{44}$	70,400	111,400	7050	190.50	190.80	190.50	70,740	111,000	0.16	191.66, ν = 0.287 Ref. [40] ${}^{†}$
${\mathrm{Fe}}_{50}{\mathrm{Ti}}_{50}$	${\mathrm{Fe}}_{46}{\mathrm{Ti}}_{54}$	69,200	10,800	6950	181.47	179.16	181.47	69,174	108,700	1.27	182.38, ν = 0.28 Ref. [52] ${}^{†}$
${\mathrm{Ti}}_{55}{\mathrm{Al}}_{45}$	${\mathrm{Ti}}_{40}{\mathrm{Al}}_{60}$	88,700	139,800	3880	166.45	165.40	166.45	88,521	139,270	0.63	160–176 Ref. [5]
${\mathrm{Ti}}_{45}{\mathrm{Al}}_{55}$	${\mathrm{Ti}}_{32}{\mathrm{Al}}_{68}$	90,800	145,700	3609	162.60	166.90	162.60	91,460	143,000	2.64	161.99, ν = 0.265 Ref. [18] ${}^{†}$
